# Role of ultra-processed foods in modulating the effect of Mediterranean diet on human and planet health—study protocol of the PROMENADE randomized controlled trial

**DOI:** 10.1186/s13063-024-08470-6

**Published:** 2024-09-30

**Authors:** Monica Dinu, Donato Angelino, Cristian Del Bo’, Mauro Serafini, Francesco Sofi, Daniela Martini

**Affiliations:** 1 Department of Experimental and Clinical Medicine, University of Florence, Florence, Italy; 2 Department of Bioscience and Technology for Food, Agriculture and Environment, University of Teramo, Teramo, Italy; 3 Department of Food, Environmental and Nutritional Sciences (DeFENS), Università degli Studi di Milano, Milan, 20133 Italy

**Keywords:** Mediterranean diet, Food processing, Human health, Sustainability

## Abstract

**Background:**

The Mediterranean diet (MD), globally recognized for its sustainability and health benefits, traditionally emphasizes the consumption of plant-based foods in raw or minimally processed forms. However, shifting lifestyles, even in Mediterranean regions, have led to an increasing consumption of ultra-processed foods (UPF). Epidemiological evidence suggests that UPF consumption may be detrimental to human health, but there is only one clinical trial on this topic which is largely debated in the scientific community. This study aims to investigate the impact of the inclusion of UPF within a Mediterranean-based dietary pattern on cardiometabolic markers, gut microbiota, and other markers of human and planet health.

**Methods:**

Fifty clinically healthy individuals showing overweight and presenting a low-to-moderate cardiovascular risk profile will be recruited for a 7-month randomized, open, cross-over dietary trial. Eligible participants will be randomly assigned to a 3-month high-UPF MD (intervention group) or a low-UPF MD (control group), with a 1-month wash-out period. Both intervention diets will have identical food group compositions, with the intervention group consuming 5 servings/day of selected UPF items, and the control group consuming raw/minimally processed items from the same food group. Blood, urine, and fecal samples, alongside food/lifestyle diaries, will be collected from each participant before and after the dietary interventions. The primary endpoint will be the change in plasma LDL-cholesterol levels from baseline. Additional markers include blood pressure, anthropometric parameters, chemical parameters, glucose and lipid-related metabolic markers, incretins, inflammatory and oxidative stress markers, fecal microbiota composition, and short-chain fatty acids. Finally, food waste production will be evaluated through specific validated food diaries. The study has been approved by the Ethical Committee of the University of Milan and the Tuscany Regional Ethics Committee of the Azienda Ospedaliera Universitaria (AOU) - Careggi, Florence.

**Discussion:**

Results from the PROMENADE study will improve knowledge about the impact of UPF consumption on human and planet health and will contribute to the scientific debate on this topic.

**Trial registration:**

ClinicalTrials.gov NCT06314932. Registered on March 13, 2024.

**Supplementary Information:**

The online version contains supplementary material available at 10.1186/s13063-024-08470-6.

## Administrative information

**Table:** 

Title {1}	Role of ultra-processed foods in modulating the effect of Mediterranean diet on human and planet health—study protocol of the PROMENADE randomized controlled trial
Trial registration {2a and 2b}	ClinicalTrials.gov identifier: NCT06314932, registered on 13/03/2024
Protocol version {3}	Protocol number 123.23, version 2.0, date 21/11/2023
Funding {4}	The project was funded by the PRIN 2022 program of the Italian Ministry of University and Research (Grant n: 2022HW2S5T)
Author details {5a}	^1^Department of Experimental and Clinical Medicine, University of Florence, Florence, Italy; ^2^Department of Bioscience and Technology for Food, Agriculture and Environment, University of Teramo, Teramo, Italy; ^3^Department of Food, Environmental and Nutritional Sciences (DeFENS), Università degli Studi di Milano, Milan, Italy
Name and contact information for the trial sponsor {5b}	Trial sponsor: Italian Ministry of University and Research Contact name: Laura Patella Address: L.go Antonio Ruberti, 1, 00153 Roma RM, Italy Telephone: + 39 0697727649 E-mail: ufficioprin@mur.gov.it
Role of sponsor {5c}	The sponsor is responsible for covering trial costs and overseeing the study’s initiation, management, and reporting. However, it does not participate in study design, data collection, management, analysis, interpretation, report writing, or the decision to submit the report for publication.

### Introduction

#### Background and rationale {6a}

The Mediterranean diet (MD) is a dietary pattern based on the traditional foods eaten in the countries overlooking the Mediterranean Sea, characterized by a substantial intake of plant-based foods and moderate consumption of animal-based products. In recent decades, the MD has emerged as one of the healthiest dietary choices, as for human and planet health [1]. Its increased adherence has demonstrated robust associations with an enhanced cardiometabolic risk profile, reduced risks of major chronic diseases (such as cardiovascular disease, diabetes, and cancer), and a noteworthy decrease in overall mortality [2, 3]. Moreover, the MD is recognized for its low environmental impact, biodiversity richness, high sociocultural food values, and positive local economic contributions [4]. Consequently, the MD has earned its place among the most sustainable diets, aligning with the imperative to shift toward more sustainable food systems and dietary patterns, as emphasized in the “Farm to Fork Strategy” [5].

Despite the considerable evidence supporting the MD, shifts in lifestyle and available time for food preparation are impacting adherence [6]. In this context, freshly prepared meals and dishes, which are the basis of traditional dietary patterns recognized to promote long and healthy lives such as MD, are increasingly replaced by ultra-processed foods (UPF). Defined within the NOVA classification as formulations derived from industrial processes, UPF encompasses products like breakfast cereals with added sugars, savory snacks, reconstituted meat products, and pre-packaged frozen dishes [7]. The global increase in UPF consumption is alarming, with reports indicating that UPF can contribute to 50–60% of the total diet in the USA, Canada, and the UK. Although Italy currently maintains a lower energy intake from UPF (approximately 10%), significant variations based on sex, age, and body mass index (BMI) have emerged, with higher consumption among overweight and obese individuals [8]. High UPF intake correlates with diets high in calories, free sugars, fat, and saturated fats, coupled with low dietary fiber [9]—a combination associated with an increased risk of cardiometabolic diseases, cerebrovascular diseases, depression, and all-cause mortality [10].

The underlying mechanisms linking UPF to adverse health effects are subjects of ongoing debate and hypothesis formulation. UPF, seemingly indicative of poor food quality, exhibit higher levels of saturated fats, added sugars, sodium, lower vitamin and fiber content, and greater energy density compared to unprocessed or minimally processed foods [9]. Alternative explanations include the presence of adverse compounds formed during industrial food processing, increased exposure to endocrine-disrupting chemicals and phthalates from industrial plastic packaging, and sensory properties contributing to delayed satiety signaling and accelerated eating rates [11–13]. Recently, there is a hypothesis suggesting that UPF may adversely impact health by modifying the gut microbiome, influencing the selection of microbes associated with inflammation-related diseases, such as cardiovascular and metabolic diseases [14].

Despite these hypotheses and the growing epidemiological evidence indicating potential harm from UPF consumption, only one clinical trial has been published thus far [15]. Hall and colleagues conducted a randomized controlled trial involving 20 adults randomized to receive either UPF or unprocessed ad libitum diets for 2 weeks. The study reported a significant increase in body weight and overall energy intake only after the UPF-rich diet. However, limitations related to the trial’s short duration and dietary intervention composition have been highlighted [16]. Furthermore, the study primarily focused on energy intake, overlooking other potential mechanisms, especially those mediated by modulation of the microbiota. Hence, the need persists for additional randomized controlled trials to delve deeper into the factors associated with the relationship between UPF consumption and human health markers, aiming to elucidate potential mechanisms. Additionally, investigating the effect of UPFs within overall healthy and balanced dietary patterns to consider the impact of the “overall diet” remains an intriguing avenue to explore.

## Objectives {7}

The PROMENADE study aims to assess whether the inclusion of UPF within a Mediterranean-based dietary pattern affects both human health and environmental markers in a group of overweight Italian subjects through a randomized, controlled, crossover trial, comparing the effects of two identical MD interventions in terms of calorie content and nutritional balance but differing in the degree of UPF consumption.

## Trial design {8}

**Fig. 1:**
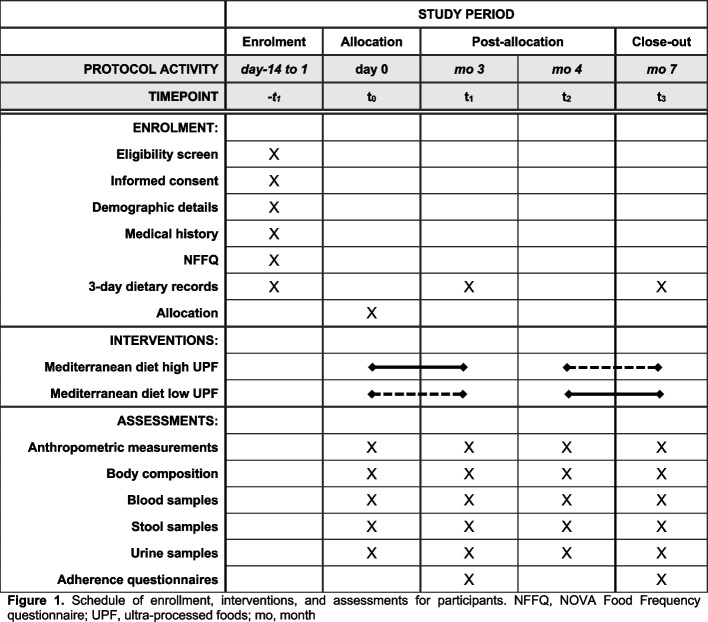
SPIRIT figure reporting the phases of the trial and data collection time points

A 7-month randomized, cross-over, two-arm, open-label, superiority trial with a 1:1 allocation ratio to either intervention or control arms will be conducted, adhering to the SPIRIT reporting guidelines (see Fig. 1 and Supplementary file 1). In the development of the study protocol, there was no direct involvement from the public or patients. Despite the acknowledged significance of patient and public engagement in research, the protocol for this study was formulated within the framework of scientific and clinical expertise.

## Methods: participants, interventions, and outcomes

### Study setting {9}

The study will be conducted by three research units (RUs): University of Milan (RU1), University of Florence (RU2), and University of Teramo (RU3). Participant recruitment and clinical evaluations will exclusively take place at RU1 and RU2. For RU1, assessments will be conducted by healthcare personnel at the licensed facility CRC-ICANS, Milan, while for RU2, evaluations will be carried out at the Unit of Clinical Nutrition of the Careggi University Hospital, Florence, Italy.

### Eligibility criteria {10}

Participants meeting the following criteria will be eligible: age > 18 years; BMI between 25.0 and 29.9 kg/m^2^, and the simultaneous presence of at least one of the following criteria, as defined by the guidelines for cardiovascular disease prevention of the European Society of Cardiology: total cholesterol levels > 190 mg/dL; LDL-cholesterol levels > 115 mg/dL; triglyceride levels > 150 mg/dL; glucose levels in the range > 111–125 mg/dL [17].

Participants will be excluded if they do not meet any of the following criteria: the presence of current serious illness or unstable conditions requiring physician supervision of diet (e.g., recent myocardial infarction, chronic liver disease, inflammatory bowel diseases, renal or digestive disorders, diabetes); pregnancy or intention to become pregnant in the next 12 months; lactation; current or recent (past 3 months) use of supplements or antibiotic therapy; current or recent (past 6 months) adoption of specific restrictive diets (e.g., low-calorie or vegetarian diets).

### Who will take informed consent? {26a}

Informed consent will be obtained by trained research personnel, including members of the study team from RU1 and RU2. These individuals will be responsible for clearly communicating the study’s objectives, procedures, potential risks, and benefits to prospective participants. Prior to enrollment, participants will have the opportunity to ask questions and receive detailed information about the trial.

### Additional consent provisions for collection and use of participant data and biological specimens {26b}

Study participants will also be asked for written informed consent to keep the biological samples for future research and to publish the data anonymously.

## Interventions

### Explanation for the choice of comparators {6b}

Our approach to comparators involves implementing two normocaloric diets with identical food group compositions, differing only in the NOVA category of 5 serving/day of foods. This design aims to investigate the impact of UPF within overall healthy and balanced dietary patterns, considering the holistic influence of the “overall diet.” The rationale behind this choice stems from an effort to address the limitations of the sole available randomized controlled trial [15]. Notably, this trial, comparing UPF with unprocessed ad libitum diets over a 2-week period, introduces potential variability in caloric intake and nutritional quality between the groups, thereby introducing confounding factors [16].

### Intervention description {11a}

The intervention group (high-UPF MD group) will consume 5 servings per day of UPF (classified by the NOVA system as NOVA 4) more than the control group. Examples include flavored yogurt, breakfast cereals with added sugar, and processed meat. In the control group, these foods will be substituted with non-ultra-processed alternatives from the same food groups (e.g., plain yogurt, breakfast cereals with no added sugar, unprocessed meat). Diets will be normalized for energy intake based on participant requirements, ensuring complete isocaloric equivalence—approximately 50–55% of energy from carbohydrates, 30–35% from total fat, and 15–20% from protein.

All participants will receive a detailed 1-week menu plan and a leaflet, developed by trial personnel considering diverse factors: (i) alignment with MD pillars and food groups [18]; (ii) adherence to Italian food habits (e.g., five meals per day, a typical sweet-oriented breakfast); (iii) market availability of food pairs within the same group but of different NOVA categories (e.g., NOVA 1 versus NOVA 4). Examples include cereal-based products (e.g., 100% cereal flakes versus cereals with added ingredients, raw pasta versus ready to eat/cook pasta), fruit and vegetables (e.g., minestrone soup made with fresh vegetables versus the canned ones), dairy (e.g., plain versus flavored yogurt), fish (e.g., fillets versus nuggets), and meat products (e.g., steaks versus cured-processed meats).

No meals or supplements will be provided. Participants will prepare their own meals or eat at a restaurant. For both diets, alcoholic beverages will be limited to one per day for women and two per day for men. Participants will be asked not to change their exercise habits during the study.

### Criteria for discontinuing or modifying allocated interventions {11b}

Participants may discontinue the intervention or withdraw from the study under the following circumstances: (1) participant’s request; (2) if the investigator determines that a participant’s health is at risk due to adverse events or the development of concomitant illness post-entry into the study.

### Strategies to improve adherence to interventions {11c}

To improve adherence to the intervention protocols, participants will receive detailed 1-week menu plans from qualified nutritionists, specifying food ingredients by weight and/or volume. Additionally, a comprehensive handout detailing the assigned diet, including possible substitutions, will be provided. We will implement recurring telephonic follow-ups to ensure ongoing support and monitor adherence to the intervention. During these follow-up sessions, participants will have the opportunity to discuss their adherence to the intervention, address any challenges encountered, and provide feedback on their dietary choices and preferences.

### Relevant concomitant care permitted or prohibited during the trial {11d}

During the trial, participants will be requested to advise if they require initiation of cortisone therapies, antidiabetic drugs, or vitamin/mineral supplements, and their continuation in the study will be evaluated accordingly. Additionally, the use of products designed for weight loss will not be permitted. If a participant requires the initiation of any of these products based on a doctor’s recommendation during the intervention period, they will be excluded from the study.

### Provisions for post-trial care {30}

Due to the nature of the study, no post-trial arrangements are required.

### Outcomes {12}

The primary endpoint will be the change in LDL-cholesterol levels from baseline. Key secondary endpoints will include changes in total cholesterol and glycemic profile, circulating levels of adipokines, inflammatory markers, oxidative stress, markers of endothelial and intestinal permeability, microbiota composition, and short-chain fatty acids (SCFAs) values after 3-month dietary intervention vs. baseline. Additionally, the study will investigate the impact of interventions on environmental constraints, such as food waste and metabolic food waste. LDL-cholesterol will be measured as a unique and established quantitative primary outcome, while the assessment of anthropometric parameters, body composition, biochemical profile, SCFAs, and gut microbiota will provide information on the cardiometabolic status of participants. The metric used for analysis will be the change in mean values from the beginning to the end of each 3-month dietary intervention.

### Participant timeline {13}

**Fig. 2:**
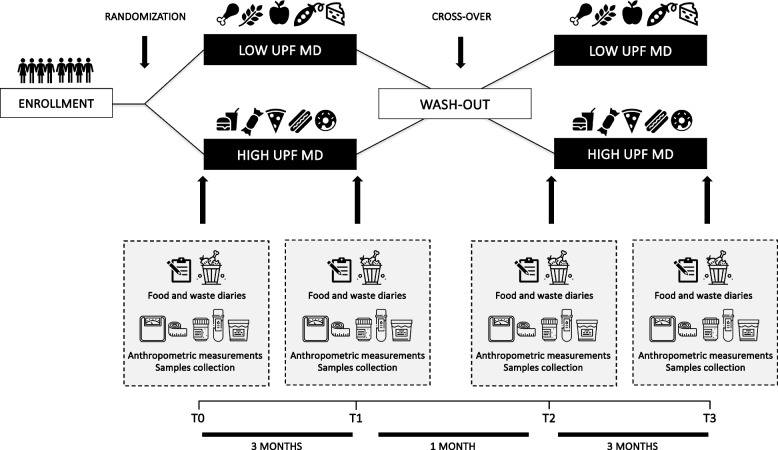
Organization of the intervention study

The study timeline is depicted in Fig. 2. Before starting the intervention, a 2-week run-in period will be implemented during which participants will be asked to complete a 3-day dietary record. Subsequently, participants will be randomly allocated into two groups: one assigned to the intervention arm and the other to the control arm. There will be four clinical evaluations of the study population: at the beginning (time 0) and at month 3 (T1) of the first dietary intervention, and at month 4 (T2) and month 7 (T3) of the second dietary intervention. At each time point, assessments of anthropometric measurements, body composition, and blood pressure will be made, and blood, stool, and urine samples will be collected.

### Sample size {14}

Sample size calculation is based on the results of a previously published study evaluating the impact of a MD intervention on human health [19]. To detect a 10% change in LDL-cholesterol levels with a statistical power of 90% (beta) and alpha set at 0.05, a randomization of 38 subjects (≥ 19 subjects in each group) is considered necessary. Considering possible dropouts, the number of participants was increased to 25 per group. Dropouts will be incorporated into the intention-to-treat analysis, including all participants initially randomized. In addition, per-protocol analysis will be conducted, focusing on those who successfully complete the study without major deviations.

### Recruitment {15}

Female and male participants will be recruited using advertisements on local media, newspapers, social media, official papers, and websites. We will also recruit from our existing database of participants and friends or relatives of the hospital and university staff.

## Assignment of interventions: allocation

### Sequence generation {16a}

A web-based online randomization procedure, free from adaptive randomization procedures, will be employed to generate the allocation sequence. The random assignment sequence will be conducted and overseen by an investigator who will not be involved in participant recruitment, ensuring an impartial and unbiased sequence generation.

### Concealment mechanism {16b}

The order of assignment will be concealed from the experimenters responsible for participant enrolment or intervention assignment. The group assignment details will be presented on a folded sheet of paper enclosed within a sealed envelope, safeguarding the integrity of the allocation sequence until the point of intervention assignment.

### Implementation {16c}

The allocation sequence for the study will be generated at RU1 and RU2 by an external researcher. Participant enrolment will be carried out by trial staff at both research units. The actual assignment of participants to interventions will be executed by the personnel directly involved in visiting the participants.

## Assignment of interventions: blinding

### Who will be blinded {17a}

Blinding of study participants and nutritionists will not be feasible due to the apparent differences between intervention diets. However, outcome measures, being less susceptible to observer influence, will be objectively assessed. Study personnel involved in participant enrolment, data collection, outcome assessment, and data analysis will be blinded to the treatment assignments. To ensure data integrity, an external researcher, independent of the research team, will enter information into the database.

### Procedure for unblinding if needed {17b}

The design is open label with participants and nutritionists not blinded so unblinding will not occur.

## Data collection and management

### Plans for assessment and collection of outcomes {18a}

Assessment and data collection at baseline and follow-up will be conducted at RU1 and RU2 by trained study staff. Subjects will undergo examination between 7:30 am and 10:30 am following a 12-h fasting period. Initially, participants will receive detailed instructions about the trial’s objectives and methodologies. The standardized baseline assessment for both groups will encompass a questionnaire addressing demographic information, risk factors, comorbidities, and the NOVA Food Frequency questionnaire (NFFQ)—a validated tool for estimating UPF consumption in the Italian population [20]. Participants will complete a 3-day weighted dietary record (two weekdays and one weekend day) to enhance the accuracy of energy and nutrient intake estimation. A dietitian, utilizing specific nutritional software linked to a country-specific food-nutrient database, will retrieve these data.

Anthropometric measurements, including height, weight, and BMI, will be recorded using a stadiometer and a professional weighing scale. Furthermore, measurements of waist and hip circumferences will be taken, with subsequent calculation of the waist-to-hip ratio. Body composition will be assessed using a bioelectrical impedance analyzer. Blood pressure will be measured with a sphygmomanometer. Blood, urine, and fecal samples will be collected at baseline and follow-up visits for subsequent analyses of biochemical, inflammatory, oxidative stress markers, and microbiota-associated markers.

At the conclusion of the visit, intervention diets will be dispensed by trial personnel. All participant sessions will maintain uniformity in duration and content. Nutritionists will be instructed not to express favoritism toward either diet or disclose personal eating habits.

### Plans to promote participant retention and complete follow-up {18b}

Strategies to enhance participant retention and follow-up completion incorporate behavior change methodologies, including self-monitoring, alongside continuous availability of study staff for dietary counseling. Upon study completion, participants will be requested to complete an adherence questionnaire, providing insights into their compliance with assigned diets, modifications made, and encountered difficulties. Adherence to the MD will be assessed using the MEDI-LITE adherence score [21] at baseline and during follow-up visits. Participants achieving ≥ 10 points on a scale ranging from 0 to 18 will be considered adherent [22]. For assessing compliance to UPF consumption, food diaries will be evaluated.

In cases where participants miss scheduled appointments, up to three phone calls and an email will be initiated before considering withdrawal from the study. Participants opting for premature discontinuation will not undergo further clinical and laboratory evaluations. The reasons for withdrawal will be documented for subsequent analysis during result interpretation. The entire study will be discontinued if observed results necessitate premature discontinuation.

### Data management {19}

All data will be systematically recorded in an electronic database. Participants will be identified solely by a unique study identification number to maintain pseudonymization, and no personally identifiable details will be stored in the database. To ensure data quality, various strategies will be employed, including meticulous recruitment, a structured and time-limited protocol, a run-in period, minimized participant burden, and the establishment of a trusting relationship between research units and participants. Additionally, double data entry will be implemented. Biological samples will be stored under optimal conditions following standard procedures. Blood samples will be aliquoted and preserved at − 20 °C for up to 5 years, with proper documentation of their usage or destruction. Preserved samples will be exclusively utilized for research purposes with the donor’s consent. Destruction of samples will be properly documented. The data will be made available upon request after publication.

### Confidentiality {27}

Identifiable details will be safeguarded through the allocation of unique study identification numbers, ensuring anonymity in data storage and analysis. Hard copies linking participant identification numbers to contact details will be stored in a locked file cabinet within a secure office. Access to this information will be restricted to key members of the research team. Participant files, source data, and related study documents will be retained for 5 years, the maximum duration allowed by the institution.

### Plans for collection, laboratory evaluation, and storage of biological specimens for genetic or molecular analysis in this trial/future use {33}

Blood, fecal (four or five scoops totaling 4 g), and urine samples will be collected at the beginning and end of each intervention phase.

#### Analysis of biochemical parameters, adipokines, and inflammatory markers (RU1, RU2, and RU3)

Blood samples will be centrifuged at 3000 rpm for 15 min to obtain serum and plasma. Samples will be aliquoted and stored at − 20 °C until analysis. Biochemical parameters (complete blood count with formula, lipid and glycemic profiles, vitamin and mineral profile) will be evaluated in all participants according to standardized routine laboratory protocols. Fasting insulin secretion capacity will be evaluated as the Homeostatic Model Assessment for Insulin Resistance (HOMA-IR) according to the related equation. Fasting plasma concentrations of ghrelin, leptin, adiponectin, resistin, visfatin, and plasminogen activator inhibitor 1 (PAI-1) will be measured using commercial enzyme-linked immunosorbent assay kits, according to the manufacturer’s instructions. Pro-(anti)-inflammatory cytokines (e.g., interleukin (IL)-1ra, IL-4, IL-10, interferon-gamma (IFN-γ), tumor necrosis factor-alpha (TNFα)) will be analyzed in plasma according to manufacturers’ instructions.

#### Analysis of oxidative stress to macromolecules (RU1, RU3)

The 5-, 8-, 12-, and 15-F2-isoprostanes will be detected and quantified in urine samples through a high-performance liquid chromatography-tandem mass spectrometry (HPLC–MS/MS) method [23]. Protein and additional markers of lipid damage (e.g., oxidized LDL) will be investigated through analysis of protein carbonyls in serum samples by using specific enzyme-linked immunosorbent assay (ELISA) assays. The evaluation of deoxyribonucleic acid (DNA) damage will employ the Comet assay, specifically focusing on hydrogen peroxide (H2O2)-induced DNA strand breaks and endogenous oxidized DNA bases, providing insights into oxidative stress [24].

#### Analysis of markers of endothelial and intestinal permeability (RU1)

Endothelial permeability will be measured through the analysis in serum samples of several markers (e.g., VE-cadherin, occludin (OCLN), and claudin-5 (CLDN-5)), while intestinal permeability will be assessed by measuring zonulin, calprotectin, and tight junction proteins in serum and fecal samples. Analysis will be performed by using ELISA assays.

#### Analysis of fecal microbiota profiles and SCFAs (RU2)

Fecal sample collection kits, including containers, will be provided to each participant. Fecal microbiota profiles and SCFAs (acetic, butyric, isobutyric, propionic, valeric, and isovaleric acids) will be evaluated. Total microbial DNA will be extracted from the faces by repeated scouring. The V3 and V4 hypervariable regions of the 16S rRNA gene for bacteria and ITS1-4 for fungi will be sequenced on the Illumina MiSeq platform, following the Illumina protocol for preparing 16S metagenomic sequencing libraries. SCFAs will be extracted using aqueous sodium hydroxide (NaOH) containing an internal standard. After extraction, an aliquot of supernatant fecal water will be derivatized with a propanol/pyridine mixture. The organic extract will be analyzed by gas chromatography-mass spectrometry (GC–MS) using deuterated internal standards and an appropriate GC Wax column.

#### Analysis of fatty acid composition of red blood cell phospholipids (RU1)

From the tube containing plasma, the buffy layer of white blood cells will be removed using a pipette. Red blood cells (RBCs) will be washed twice in an equal volume of a physiologic solution (0.9% NaCl, w/v). Two aliquots (0.5 mL) of RBCs will be stored at − 80 °C until the analysis. Extraction of RBC phospholipids will be carried out in accordance with the method previously described by Simonetti et al. [25]. The FA composition of RBCs will be obtained by GC analysis following the method described by Ackman [26].

#### Measurement of food waste and metabolic food waste (RU1, RU3)

Food waste will be monitored by a specific food diary [27]. Subjects will be asked to fill a 3-day weighed food waste diary for each intervention arm, by registering type and weighed amount of waste. Waste will be quantified not only as a net amount of food waste (grams per week of total waste and for each food group) but also performing a nutritional and environmental assessment of food wasted [28].

The Metabolic Food Waste indicator [MFW (kg of food)] corresponds to the amount of food leading to an excess of body fat and its impact on the environment expressed as carbon [MFW (kgCO2 eq)], water [MFW(× 10 L)], and land footprint [MFW(× 10 m^2^)] [29]. Calculations will be applied in agreement with our previous publication [29] with few modifications, including data from new databases recently developed [28]. Briefly, the dietary intakes of volunteers will be analyzed and aggregated based on similar macronutrient composition. Then, the energy contribution of each food/food category to the daily energy intake will be calculated. Fruits and vegetables will be excluded because they are low energy dense foods not contributing to obesity. Then, anthropometric measurements relative to body fat mass will be considered. The difference between individual and average body fat mass will be multiplied for energy content of 1 kg of body fat to reach the total energy from exceeding body fat and distributed among the different foods according to their percentage contribution to total energy intake. The acquired data will allow us to calculate the carbon, water, and land footprints.

#### Calculation of the inflammatory index of foods (RU3)

The pro-(anti)-inflammatory effect of the tested food samples will be assessed after a simulated in vitro human digestion and, consequently, their exposure in a human culture cell-based in vitro model will be assessed, with the production of a small database of results for NII development. Aliquots of pairs of foods belonging to the same food group but of different NOVA categories (e.g., NOVA 1 versus NOVA 4) will be processed, i.e., washing, cleaning, cutting, mincing, and undergo cooking procedures, to be used for a human simulated in vitro digestion process, following the protocol by Minekus et al. [30]. In detail, time-dependent subsequent oral, gastric, and intestinal phases of digestion will be applied by mixing foods with solutions of digestive enzymes and buffer solutions for the digestion of carbohydrates, lipids, and proteins. Digested samples obtained during the time will be aliquoted and stored at − 20 °C till analysis.

Concerning the anti-(pro)-inflammatory effects of such digested foods, the digested samples will be incubated with innate immune cells (THP-1). Three concentrations of digesta will be used based on preliminary experiments, to identify a dose response effect, assessing the inflammatory response in the supernatants by ELISA assays after 24 h [31]. The inflammatory response will be measured in terms of 4 pro-inflammatory and anti-inflammatory factors’ production, such as inflammatory chemokines (IL-8), inflammatory cytokines (TNFα), and anti-inflammatory cytokines (IL-10, transforming growth factor-β (TGF-β)) [31]. The inflammatory response of the THP-1 to digested food samples will be compared to the response to a prototypical inflammatory stimulus (i.e., lipopolysaccharides (LPS)), used as reference control. To define the pro or anti-inflammatory unbiased effects due to digested samples and not to endotoxin contamination, the amount of endotoxin (e.g., LPS) in the digested samples will be evaluated by the chromogenic Limulus amoebocyte lysate assay [32, 33].

## Statistical methods

### Statistical methods for primary and secondary outcomes {20a}

Statistical analyses will be performed using SPSS software for Macintosh (SPSS Inc., Chicago, IL, USA). A *P* value < 0.05 will be considered statistically significant. Data will be analyzed using intention-to-treat and on-treat procedures. The primary outcome (change in body weight) will be analyzed within each group using Student’s *t*-test for paired data. The difference in absolute change (mean value at baseline subtracted from the mean value after the intervention for each subject) will be estimated using the Student’s *t*-test for independent data. Evaluation of distributions and checking for outliers will be performed using histograms and box plots. If appropriate, variables will be logarithmized to normalize the distributions of data. Continuous variables that follow a normal distribution will be summarized using the mean and standard deviations. Categorical variables will be presented in terms of frequencies and percentages. After testing the regression assumption, a general linear model for repeated measurements with adjustments for possible confounding factors will be run to compare the effects of different interventions. Data for the general linear model will be reported as geometric means and 95% confidence intervals. The same analyses will be performed for secondary outcomes. Microbial alpha (richness, Simpson’s, Gini-Simpson’s, inverse Simpson and Shannon’s indices, evenness and dominance) and beta (weighted UniFrac and Bray–Curtis dissimilarity) diversity measures will be assessed using QIIME (v. 1.9).

### Interim analyses {21b}

No interim analysis will be conducted.

### Methods for additional analyses (e.g., subgroup analyses) {20b}

Subgroup analyses will be performed to analyze possible differences in the changes, according to specific characteristics of the study population (age and sex).

### Methods in analysis to handle protocol non-adherence and any statistical methods to handle missing data {20c}

Before starting the data analysis, the level and possible causes of missing data will be investigated using appropriate tables. This information will be used to determine whether the level and type of missing data have the potential to introduce bias into analysis results or substantially reduce the precision of the estimates for the proposed statistical methods. Sensitivity analyses will be performed, based on the assumption that the missing outcomes are the worst or best possible in the different randomization groups. If these show that the conclusions may differ based on the missing values, further multiple imputation will be performed for the missing values. These analyses will consider the results of any losses at follow-up to the extent that they relate to differences in the measured variables (i.e., under the assumption of random missingness).

### Plans to give access to the full protocol, participant-level data, and statistical code {31c}

The datasets analyzed during the current study and statistical code are available from the corresponding author on reasonable request, as is the full protocol.

## Oversight and monitoring

### Composition of the coordinating center and trial steering committee {5d}

Regular monitoring of this study will be conducted by both the research team and the local Institutional Review Board. Given the study’s limited objectives, short duration, and the low-risk nature of the intervention, the establishment of a data monitoring committee and a trial steering committee was deemed unnecessary. A management board, chaired by the principal investigator (PI) and including one representative from each RU, will monitor the progress of activities toward the targets. The board will meet at least every 6 months and whenever needed depending on the progress of scientific activities, with detailed reports about the findings and discussions on the putative critical points. The board will be in charge of the definition of the deadlines related to the different steps of the project as well as to the relative reports, to be shared with all the consortium participants. Finally, annual reports on activities and financial matters will be prepared.

### Composition of the data monitoring committee, its role and reporting structure {21a}

The research group, appointed by the principal investigator, comprises additional two study coordinators, two physicians, six nutritionists, and two biostatisticians.

### Adverse event reporting and harms {22}

Adverse events, such as unfavorable and undesirable signs, symptoms, abnormal laboratory results, or illnesses temporally associated with the intervention diet, will be collected from the time of randomization until the final 7-month follow-up visit for each participant, whether or not they are considered related to the intervention study. All adverse events will be followed up until their resolution.

### Frequency and plans for auditing trial conduct {23}

The team will convene regularly, typically monthly, to comprehensively assess ongoing trial progress. This includes a thorough review of study advancements, recruitment rates (actual versus predicted), data quality and return rates, protocol amendments, and general research study matters. The principal investigator will oversee these meetings, developing the agenda and serving as the study’s primary contact. At the halfway point and conclusion of the study, the protocol team will furnish the local Institutional Review Board with a comprehensive monitoring report, encompassing a review of activities, progress, challenges, and any pertinent issues of concern.

### Plans for communicating important protocol amendments to relevant parties (e.g., trial participants, ethical committees) {25}

Any changes to the protocol and information provided to participants will be submitted to the Ethics Committee for approval prior to implementation.

## Dissemination plans {31a}

The study results will be shared through several channels. Investigators will present findings at both national and international scientific conferences, and a dedicated webinar for Italian nutritionists will elucidate UPF consumption within the MD. Scientific publications in peer-reviewed journals are part of the dissemination plan. The strategy encompasses public involvement, featuring a consumer event, participation in “Researchers’ Night,” and the creation of informative materials. An online and social media campaign will enhance visibility, engaging a wide audience, including stakeholders and the public.

## Discussion

The scientific literature on UPF reports divergent perspectives, mainly revolving around the interpretation of observational data and the need for rigorous interventional studies. Advocates for public health policies restricting UPF intake and incorporating UPF guidelines into dietary recommendations rely on epidemiological evidence linking their consumption to adverse health outcomes [34–36]. In contrast, part of the scientific community calls for a cautious interpretation, citing the inherent limitations of observational studies and the need for randomized controlled trials to establish causality and clarify underlying mechanisms [37–39]. Furthermore, there is acknowledgment of the heterogeneity within UPF, with recent data suggesting that not all UPF pose equal health risks [40, 41].

In this context, the PROMENADE study represents a pioneering effort as the first clinical trial investigating UPF consumption within the framework of a MD. Its potential impact extends across scientific, industrial, and social dimensions, serving as a significant contribution to the ongoing discourse on nutrition, health, and sustainability. Scientifically, PROMENADE is expected to contribute to our understanding of the relationship between food processing and health in the context of a dietary intervention based on the MD. The results have the potential to provide insights into the degree to which food processing, particularly UPF, influences the impact of the MD on human health. From an industrial perspective, insights from the study may guide the formulation of UPF with improved nutritional profiles, aligning convenience with health considerations within a sustainable food model. At a social level, dissemination of results could enhance informed dietary choices on a broader scale.

Beyond its implications for human health, PROMENADE also holds relevance for planetary health. UPF consumption is associated with unsustainable agricultural practices and excessive packaging waste, contributing to environmental degradation [42–44]. In contrast, the MD aligns more closely with sustainable food production practices, boasting a lower environmental footprint [4]. By examining the combined effects of UPF consumption and adherence to a Mediterranean-based dietary pattern on both human health and the environment, the trial can inform dietary recommendations that promote individual health while minimizing the ecological impact of food production.

Strengths of the study include its randomized controlled trial design, enabling rigorous assessment of causal relationships. Randomization minimizes confounding factors, enhancing internal validity and allowing for more reliable conclusions. Additionally, comprehensive outcome measures, including body weight and composition, metabolic markers, and gut microbiota composition, provide a holistic understanding of the potential impacts of dietary interventions. Main limitations include study duration and sample size, which may challenge generalizability, and reliance on self-reported data that introduces the possibility of recall bias. Nevertheless, despite these limitations, the PROMENADE study holds promise in advancing our understanding of the impact of UPF consumption on both human and planetary health. By contributing substantively to the ongoing scientific discourse on this critical topic, it has the potential to inform future research endeavors and public health initiatives.

## Trial status

The study has received all necessary regulatory approvals. The currently approved version of the protocol is 2.0 (version date 21/11/2023). Recruitment will begin in April 2024 and the completion date is scheduled for April 2025.

## Supplementary Information

Supplementary Material 1.

## Data Availability

Access to the final trial dataset will be restricted to the research team to ensure confidentiality and privacy protection for participants. The principal investigator will be responsible for safeguarding participants’ privacy, and data, along with source documents, will be stored securely for potential monitoring or inspection by the Ethics Committee. Upon completion of the study, participants have the option to request a copy of the study results from the principal investigator, promoting transparency and participant engagement. The final report will adhere to the CONSORT 2010 guidelines, ensuring comprehensive reporting standards. To contribute to the scientific community’s knowledge, study results will be submitted for publication in a peer-reviewed journal. Additionally, plans for dissemination at national and international conferences will be collaboratively discussed with researchers before implementation, promoting widespread sharing of findings.

## Abbreviations

BMI: Body mass index
DNA: Deoxyribonucleic acid
ELISA: Enzyme-linked immunosorbent assay
FA: Fatty acid
GC-MS: Gas chromatography-mass spectrometry
H2O2: Hydrogen peroxide
HOMA-IR: Homeostatic Model Assessment for Insulin Resistance
HPLC–MS/MS: High-performance liquid chromatography-tandem mass spectrometry
ICMJE: International Committee of Medical Journal editors
IL: Interleukin
LDL: Low-density lipoprotein
LPS: Lipopolysaccharides
MD: Mediterranean diet
MFW: Metabolic Food Waste indicator
NaOH: Sodium hydroxide
NFFQ: NOVA Food Frequency questionnaire
PAI-1: Plasminogen activator inhibitor 1
PI: Principal investigator
rRNA: Ribosomal ribonucleic acid
RBC: Red blood cell
RU: Research unit
SCFA: Short-chain fatty acids
SPIRIT: Standard Protocol Items: Recommendations for Interventional Trials
TGF-β: Transforming growth factor-β
TNFα: Tumor necrosis factor-alpha
UPF: Ultra-processed foods
